# Redetermination of 4-(dimethyl­amino)­pyridinium tribromide

**DOI:** 10.1107/S1600536810030369

**Published:** 2010-08-11

**Authors:** Seik Weng Ng

**Affiliations:** a Department of Chemistry, University of Malaya, 50603 Kuala Lumpur, Malaysia

## Abstract

In the title salt, C_7_H_11_N_2_^+^·Br_3_^−^, the essentially planar cation (r.m.s. deviation = 0.006 Å) forms an N—H⋯Br hydrogen bond to one of the Br atoms of the almost linear anion [Br—Br—Br = 179.31 (2)°]. The crystal studied was found to be a racemic twin. The whole-mol­ecule disorder of the cation and anion about a twofold rotation axis described earlier [Ng (2009). *Acta Cryst.* E**65**, o1276] is an artifact of halving one of the axes of the ortho­rhom­bic unit cell.

## Related literature

For the refinement based on a unit cell half as large, see: Ng (2009 ▶).
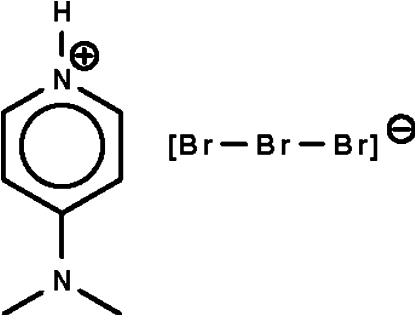


## Experimental

### 

#### Crystal data

C_7_H_11_N_2_^+^·Br_3_^−^
*M* *_r_* = 362.91
Orthorhombic, 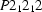

*a* = 14.7253 (2) Å
*b* = 17.6696 (3) Å
*c* = 4.1689 (1) Å
*V* = 1084.71 (4) Å^3^
*Z* = 4
Mo *K*α radiation
μ = 11.11 mm^−1^
*T* = 100 K
0.20 × 0.15 × 0.10 mm

#### Data collection

Bruker SMART APEX CCD diffractometer
Absorption correction: multi-scan (*SADABS*; Sheldrick, 1996 ▶) *T*_min_ = 0.215, *T*_max_ = 0.403
10364 measured reflections
2502 independent reflections
2300 reflections with *I* > 2σ(*I*)
*R*_int_ = 0.029

#### Refinement

*R*[*F* ^2^ > 2σ(*F* ^2^)] = 0.019
*wR*(*F* ^2^) = 0.040
*S* = 0.98
2502 reflections
116 parameters
H atoms treated by a mixture of independent and constrained refinement
Δρ_max_ = 0.38 e Å^−3^
Δρ_min_ = −0.31 e Å^−3^
Absolute structure: Flack (1983 ▶), 999 Friedel pairs
Flack parameter: 0.51 (2)

### 

Data collection: *APEX2* (Bruker, 2009 ▶); cell refinement: *SAINT* (Bruker, 2009 ▶); data reduction: *SAINT*; program(s) used to solve structure: *SHELXS97* (Sheldrick, 2008 ▶); program(s) used to refine structure: *SHELXL97* (Sheldrick, 2008 ▶); molecular graphics: *X-SEED* (Barbour, 2001 ▶); software used to prepare material for publication: *publCIF* (Westrip, 2010 ▶).

## Supplementary Material

Crystal structure: contains datablocks global, I. DOI: 10.1107/S1600536810030369/hb5583sup1.cif

Structure factors: contains datablocks I. DOI: 10.1107/S1600536810030369/hb5583Isup2.hkl

Additional supplementary materials:  crystallographic information; 3D view; checkCIF report

## Figures and Tables

**Table 1:** Hydrogen-bond geometry (Å, °)

*D*—H⋯*A*	*D*—H	H⋯*A*	*D*⋯*A*	*D*—H⋯*A*
N1—H1⋯Br1	0.92 (3)	2.41 (3)	3.323 (2)	171 (3)

**Fig. 1.**
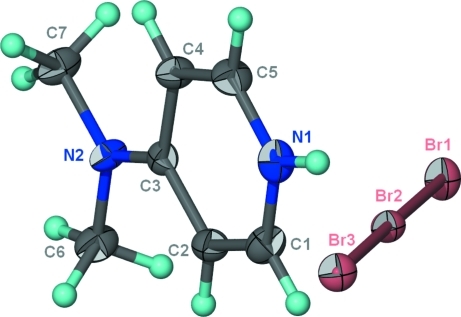
The molecular structure of (I) at the 70% probability level; hydrogen atoms are drawn as spheres of arbitrary radius.

## supplementary crystallographic information

### Comment

Dimethylaminopyridinium tribromide (I) was refined as a whole-molecule-disordered cation and anion that was disordered about a crystallographic twofold rotation axis (Ng, 2009) in the orthorhombic *P*222_1_ space group [unit cell parameters 4.1688 (1), 8.8349 (2), 14.7255 (4) Å]. The automatic cell-searching program had, in fact, missed some weaker reflections, so that the true *b*-axis should be doubled, so that the space group would be *P*22_1_2_1 _[4.1689 (1), 17.6696 (3), 14.7253 (2) Å]. In the standard *P*2_1_2_1_2 setting, the structure refines smoothly, without disorder, to a final *R* index of 0.019 (Fig. 1). The disorder is an artifact of halving one of the axis, and a chemically reasonable model coincidentally arose owing to the nature of both the planar cation and linear anion.

### Experimental

The diffraction measurements were those used in the previous study (Ng, 2009). Measurements on another different specimen gave the same refinement results, especially with respect to the 0.5 Flack parameter.

### Refinement

Carbon-bound H-atoms were placed in calculated positions (C—H 0.95 Å for the aromatic H-atoms and 0.98 Å for the methyl H-atoms) and were included in the refinement in the riding model approximation, with *U*(H) set to 1.2 to 1.5*U*(C).

The ammonium H-atom was located in a difference Fourier map, and was refined without a restraint.

The structure is a racemic twin; the Flack parameter was refined on 999 Friedel pairs.

### Crystal data

**Table:**

C_7_H_11_N_2_^+^·Br_3_^−^	*F*(000) = 688
*M**_r_* = 362.91	*D*_x_ = 2.222 Mg m^−^^3^
Orthorhombic, *P*2_1_2_1_2	Mo *K*α radiation, λ = 0.71073 Å
Hall symbol: P 2 2ab	Cell parameters from 4074 reflections
*a* = 14.7253 (2) Å	θ = 2.7–28.3°
*b* = 17.6696 (3) Å	µ = 11.11 mm^−^^1^
*c* = 4.1689 (1) Å	*T* = 100 K
*V* = 1084.71 (4) Å^3^	Block, colorless
*Z* = 4	0.20 × 0.15 × 0.10 mm

### Data collection

**Table:**

Bruker SMART APEX CCD diffractometer	2502 independent reflections
Radiation source: fine-focus sealed tube	2300 reflections with *I* > 2σ(*I*)
graphite	*R*_int_ = 0.029
ω scans	θ_max_ = 27.5°, θ_min_ = 1.8°
Absorption correction: multi-scan (*SADABS*; Sheldrick, 1996)	*h* = −19→19
*T*_min_ = 0.215, *T*_max_ = 0.403	*k* = −22→22
10364 measured reflections	*l* = −5→5

### Refinement

**Table:**

Refinement on *F*^2^	Secondary atom site location: difference Fourier map
Least-squares matrix: full	Hydrogen site location: inferred from neighbouring sites
*R*[*F*^2^ > 2σ(*F*^2^)] = 0.019	H atoms treated by a mixture of independent and constrained refinement
*wR*(*F*^2^) = 0.040	*w* = 1/[σ^2^(*F*_o_^2^) + (0.0204*P*)^2^] where *P* = (*F*_o_^2^ + 2*F*_c_^2^)/3
*S* = 0.98	(Δ/σ)_max_ = 0.001
2502 reflections	Δρ_max_ = 0.38 e Å^−^^3^
116 parameters	Δρ_min_ = −0.31 e Å^−^^3^
0 restraints	Absolute structure: Flack (1983), 999 Friedel pairs
Primary atom site location: structure-invariant direct methods	Flack parameter: 0.51 (2)

### Fractional atomic coordinates and isotropic or equivalent isotropic displacement parameters (Å^2^)

**Table:**

	*x*	*y*	*z*	*U*_iso_*/*U*_eq_	
Br1	0.422049 (17)	0.112471 (15)	0.72575 (7)	0.01934 (7)	
Br2	0.261320 (17)	0.120225 (14)	0.47003 (7)	0.01501 (7)	
Br3	0.106438 (17)	0.127359 (14)	0.23133 (7)	0.01826 (7)	
N1	0.60669 (15)	0.13040 (13)	0.2738 (6)	0.0196 (5)	
H1	0.553 (2)	0.1299 (18)	0.387 (8)	0.038 (10)*	
N2	0.85347 (15)	0.12901 (12)	−0.1849 (6)	0.0162 (5)	
C1	0.6420 (2)	0.06411 (16)	0.1723 (7)	0.0218 (7)	
H1A	0.6095	0.0185	0.2093	0.026*	
C2	0.72305 (19)	0.06148 (15)	0.0185 (7)	0.0182 (6)	
H2	0.7468	0.0143	−0.0520	0.022*	
C3	0.77231 (17)	0.12906 (14)	−0.0370 (6)	0.0148 (5)	
C4	0.73201 (19)	0.19758 (15)	0.0729 (7)	0.0179 (6)	
H4	0.7621	0.2445	0.0396	0.021*	
C5	0.65084 (18)	0.19580 (14)	0.2248 (8)	0.0200 (6)	
H5	0.6246	0.2418	0.2982	0.024*	
C6	0.89406 (19)	0.05789 (14)	−0.2953 (8)	0.0221 (7)	
H6A	0.9021	0.0237	−0.1124	0.033*	
H6B	0.9532	0.0683	−0.3936	0.033*	
H6C	0.8540	0.0341	−0.4538	0.033*	
C7	0.90234 (17)	0.19907 (14)	−0.2435 (9)	0.0215 (6)	
H7A	0.8653	0.2324	−0.3782	0.032*	
H7B	0.9597	0.1879	−0.3532	0.032*	
H7C	0.9150	0.2242	−0.0387	0.032*	

### Atomic displacement parameters (Å^2^)

**Table:**

	*U*^11^	*U*^22^	*U*^33^	*U*^12^	*U*^13^	*U*^23^
Br1	0.01273 (13)	0.02577 (14)	0.01951 (15)	−0.00074 (11)	0.00148 (13)	0.00173 (13)
Br2	0.01278 (13)	0.01445 (12)	0.01779 (13)	−0.00072 (11)	0.00346 (11)	−0.00043 (11)
Br3	0.01313 (13)	0.02064 (13)	0.02101 (14)	−0.00046 (11)	0.00112 (13)	−0.00002 (14)
N1	0.0121 (11)	0.0263 (12)	0.0204 (13)	0.0014 (10)	0.0026 (12)	0.0015 (13)
N2	0.0133 (11)	0.0125 (10)	0.0228 (13)	0.0005 (9)	0.0026 (10)	0.0024 (10)
C1	0.0188 (16)	0.0211 (15)	0.0255 (18)	−0.0030 (13)	0.0007 (14)	0.0025 (12)
C2	0.0195 (15)	0.0157 (13)	0.0196 (16)	0.0012 (11)	0.0001 (15)	−0.0015 (12)
C3	0.0135 (13)	0.0170 (12)	0.0140 (12)	0.0004 (12)	−0.0034 (12)	0.0014 (12)
C4	0.0154 (14)	0.0157 (13)	0.0225 (16)	−0.0005 (12)	0.0018 (14)	−0.0014 (11)
C5	0.0177 (14)	0.0186 (13)	0.0236 (17)	0.0051 (11)	0.0038 (17)	−0.0030 (14)
C6	0.0183 (15)	0.0178 (13)	0.0303 (19)	0.0030 (12)	0.0047 (18)	0.0008 (14)
C7	0.0141 (14)	0.0179 (13)	0.0323 (18)	−0.0011 (11)	0.0072 (18)	0.0024 (15)

### Geometric parameters (Å, °)

**Table:**

Br1—Br2	2.5994 (4)	C2—H2	0.9500
Br2—Br3	2.4915 (4)	C3—C4	1.424 (4)
N1—C5	1.342 (3)	C4—C5	1.353 (4)
N1—C1	1.350 (4)	C4—H4	0.9500
N1—H1	0.92 (3)	C5—H5	0.9500
N2—C3	1.345 (3)	C6—H6A	0.9800
N2—C7	1.453 (3)	C6—H6B	0.9800
N2—C6	1.466 (3)	C6—H6C	0.9800
C1—C2	1.356 (4)	C7—H7A	0.9800
C1—H1A	0.9500	C7—H7B	0.9800
C2—C3	1.416 (4)	C7—H7C	0.9800
			
Br3—Br2—Br1	179.314 (16)	C5—C4—H4	120.0
C5—N1—C1	120.9 (2)	C3—C4—H4	120.0
C5—N1—H1	120 (2)	N1—C5—C4	121.3 (3)
C1—N1—H1	119 (2)	N1—C5—H5	119.4
C3—N2—C7	121.1 (2)	C4—C5—H5	119.4
C3—N2—C6	120.5 (2)	N2—C6—H6A	109.5
C7—N2—C6	118.4 (2)	N2—C6—H6B	109.5
N1—C1—C2	121.2 (3)	H6A—C6—H6B	109.5
N1—C1—H1A	119.4	N2—C6—H6C	109.5
C2—C1—H1A	119.4	H6A—C6—H6C	109.5
C1—C2—C3	119.9 (3)	H6B—C6—H6C	109.5
C1—C2—H2	120.0	N2—C7—H7A	109.5
C3—C2—H2	120.0	N2—C7—H7B	109.5
N2—C3—C2	122.0 (2)	H7A—C7—H7B	109.5
N2—C3—C4	121.2 (2)	N2—C7—H7C	109.5
C2—C3—C4	116.8 (2)	H7A—C7—H7C	109.5
C5—C4—C3	119.9 (3)	H7B—C7—H7C	109.5
			
C5—N1—C1—C2	0.1 (5)	C1—C2—C3—N2	179.1 (3)
N1—C1—C2—C3	0.2 (4)	C1—C2—C3—C4	−0.6 (4)
C7—N2—C3—C2	179.1 (3)	N2—C3—C4—C5	−179.1 (3)
C6—N2—C3—C2	0.1 (4)	C2—C3—C4—C5	0.6 (4)
C7—N2—C3—C4	−1.2 (4)	C1—N1—C5—C4	−0.1 (5)
C6—N2—C3—C4	179.8 (3)	C3—C4—C5—N1	−0.3 (5)

### Hydrogen-bond geometry (Å, °)

**Table:**

*D*—H···*A*	*D*—H	H···*A*	*D*···*A*	*D*—H···*A*
N1—H1···Br1	0.92 (3)	2.41 (3)	3.323 (2)	171 (3)
